# Prevalence of dysphagia following posterior fossa tumor resection: a systematic review and meta‑analysis

**DOI:** 10.1186/s12885-024-12656-1

**Published:** 2024-07-25

**Authors:** Yuyu Duan, Yueli Wang, Xiaowei Zhang, Jingjuan Huang, Zhihuan Zhou, Qinqin Zhao

**Affiliations:** 1 State Key Laboratory of Oncology in South China, Collaborative Innovation Center for Cancer Medicine, Guangdong Key Laboratory of Nasopharyngeal Carcinoma Diagnosis and Therapy, Sun Yat-sen University Cancer Center, Guangzhou, Guangdong China; 2 Department of Neurosurgery, Sun Yat-sen University Cancer Center, 651 Dongfeng Road East, Guangzhou, 510060 Guangdong PR China; 3 Operating Room, The First Affiliated Hospital of Guangzhou Medical University, Guangzhou, China

**Keywords:** Dysphagia, Posterior fossa tumor, Meta-analysis, Systematic review

## Abstract

**Objective:**

Dysphagia is common in individuals who have undergone posterior fossa tumor (PFT) resection and negatively impacts on the individual’s quality of life, nutritional status, and overall health. We aimed to quantitatively synthesize data from studies of the prevalence of dysphagia following PFT resection.

**Methods:**

PubMed, Web of Science, the Cochrane Library, Embase, China National Knowledge Infrastructure (CNKI), Wanfang database, and VIP database were searched for case-control and cross-sectional studies that evaluated the prevalence of dysphagia after PFT surgery. Meta-analyses were performed to determine the prevalence of dysphagia. Subgroup and meta-regression analyses were performed to determine the sources of heterogeneity among the studies.

**Results:**

A total of 22 studies were included, involving 20,921 cases. A meta-analysis of the random-effects model showed that the pooled global prevalence of dysphagia following PFT resection was 21.7% (95% confidence interval: 16.9–26.6). The subgroup and meta-regression analyses demonstrated that participant age (*P* < 0.001), assessment methods (*P* = 0.004), and geographical region of the study participants (*P* = 0.001) were sources of heterogeneity among the studies.

**Conclusions:**

Dysphagia has a high prevalence following PFT resection. Individuals with PFTs who are at a high risk for dysphagia should be identified early through screening. Multidisciplinary diagnosis and treatment of dysphagia are required to improve the outcomes in the early stages after PFT resection.

**Supplementary Information:**

The online version contains supplementary material available at 10.1186/s12885-024-12656-1.

## Introduction

The posterior fossa is situated below the cerebellar tentorium and above the foramen magnum, which encompasses the fourth ventricle and cerebellum [1]. Although posterior fossa tumors (PFTs) are less commonly observed in adults [2], they are the most common solid tumors in the pediatric population, occurring at an incidence rate of 2–3.5 per 100,000 individuals [3, 4]. Microsurgery remains the preferred treatment for PFTs, particularly in cases involving malignancy, facial nerve dysfunction, hearing deterioration, and obstructive hydrocephalus [5].

The posterior fossa contain complex and vital structures [6], such as the cerebellum and brainstem, which play critical roles in the precise and efficient execution of speech and swallowing movements [7]. Surgical interventions for PFTs can lead to various serious adverse events, including posterior fossa syndrome/cerebellar mutism syndrome, muscle weakness, sensory changes, imbalance, cognitive dysfunction, and dysphagia [8]. Dysphagia, defined as impairment of the swallowing process, can occur if there is a malfunction with any part of the swallowing mechanism [9]. The two most common causes of dysphagia are (1) neurologic or anatomic injury of the cerebral cortex, brainstem, or cranial nerves (IX–XII) related to swallowing and (2) direct injury to the muscles of swallowing [9]. Anatomically adjacent to the site of PFTs, crucial structures such as brainstem, cerebellar tissue, or cranial nerves are prone to injury as a result of surgical removal, resulting in secondary focal neurological deficits [10]. In addition, dysphagia is also seen in cerebellar mutism or posterior fossa syndrome, which is a syndrome of clinical features after posterior fossa surgery characterized by mutism, emotional lability, hypotonia, and ataxia [11, 12]. In a study of 27 children with a PFT by Mei and colleagues, the incidence of postsurgical dysphagia was lower compared with a previous report that included only children with mutism after resection of PFTs [7, 13]. A possible reason for the discrepancy between these two studies relates to the presence of cerebellar mutism syndrome. Therefore, dysphagia is one of the commonly reported risks of surgical PFTs resection.

In patients with PFTs, dysphagia requires special attention as it can result in aspiration-induced lung diseases or malnutrition [14–16], leading to prolonged hospital stays and even mortality [9, 17]. The estimated annual cost of dysphagia in the US is $4–7 billion [18]. Furthermore, dysphagia has a profound negative impact on the emotional and mental well-being of patients, significantly affecting their quality of life [19].

Given the significant improvements in survival rates of PFTs resection in recent years, therapeutic strategies for the management of postoperative dysphagia and enhancement of the functional well-being have received increasing attention. Consequently, numerous studies have investigated the prevalence of dysphagia following PFT removal. However, no detailed and systematic meta-analysis has been conducted on the prevalence of dysphagia following PFT removal. While several studies have focused on patients with PFTs and reported the frequencies of dysphagia ranging from 33% to approximately 70% [1, 7, 11, 20], Ward et al. conducted a retrospective cross-sectional study involving 17,281 patients who underwent surgery for vestibular schwannoma, reporting a prevalence of dysphagia of 2.6% [21]. Studies of dysphagia following PFT removal had similar limitations, including small sample sizes and collection of data from a single healthcare setting. Consequently, there is limited information available on the global prevalence of dysphagia following PFT removal. Therefore, the aim of this study was to evaluate the prevalence of dysphagia following PFT removal, which could provide researchers and clinicians with the most recent information for the early detection, effective prevention, and adequate monitoring of postoperative dysphagia.

## Methods

### Protocol and registration

The review was prospectively registered in the PROSPERO database (registration no.: CRD42023441428).

### Literature search

A comprehensive literature search was conducted using various databases, including PubMed, Web of Science, The Cochrane Library, Embase, China National Knowledge Infrastructure, Wanfang database, and VIP database. Articles published from database inception until March 31, 2023 were searched. The search strategy involved a combination of MeSH (Medical Subject Headings) and free-text terms. The key concepts used in the search were “dysphagia,” “posterior fossa tumors,” and “prevalence.” For each database, a tailored search query was developed to account for its unique characteristics. Supplementary material S1 provide a summary of the search strategy. In addition, manual searches of the reference lists of the retrieved articles were performed to identify any additional published studies relevant to the topic.

### Inclusion and exclusion criteria

The review included observational studies, including case-control, cross-sectional, and longitudinal cohort studies. Studies that evaluated postoperative dysphagia in children and/or adult patients diagnosed with a PFT were included. We excluded reviews, meta-analyses, comments, meeting summaries, letters, duplicate literature, articles in languages other than English and Chinese, animal or cell studies, studies with incomplete information or missing data that could not be obtained through other sources, and studies focusing on patients who underwent posterior fossa surgery other than PFT resection.

### Study selection and data extraction

Two investigators independently extracted the relevant information from articles that fulfilled the eligibility criteria and entered it into Excel worksheets. Disagreements between the investigators were resolved through consensus or discussion with a third investigator. The investigators extracted the basic information related to the study (name of the first author, publication year, region of the study population, and age range) and the main study characteristics (study design, total sample size, number of patients with dysphagia, assessment method of dysphagia, tumor site, and tumor types).

### Quality assessment

Two investigators independently assessed the quality of the included studies using the Joanna Briggs Institute critical appraisal checklist (Supplementary Material S2). Disagreements in the quality assessments between the investigators were resolved through consensus. The scale includes nine items, with each item scored 0 (not qualified), 1 (mentioned but not described in detail), or 2 (detailed, comprehensive, and correct description). The sum of the domain scores (the highest possible score was 18), along with the percentage of the highest score, was computed for each study. A score > 70% of the maximum possible score denoted a high quality of the study.

### Statistical analysis

Stata software (version 15.0; Stata Corp., College Station, TX, USA) was used to perform the meta-analyses, while the Review Manager software (RevMan 5.3; Cochrane Collaboration, Oxford, United Kingdom) was used to estimate the risk of bias. The primary outcome was the prevalence of dysphagia, recorded using basic descriptors (effect size [95% confidence interval]). Heterogeneity was evaluated based on the *I*^2^ values; *I*^2^ values > 75% indicated high heterogeneity among studies. The heterogeneity *P*-values were used to evaluate the potential heterogeneity among studies, with the level of significance set at 0.10. In cases of *I*^2^ ≤ 50%, which indicated low heterogeneity among studies, a fixed-effects model was used for analysis; otherwise, a random-effects model was used. If heterogeneity existed, subgroup analyses of the included studies were conducted according to the study design, publication year, age, assessment method of dysphagia, tumor sites, tumor types, and geographical region of the study population (such as North America, Europe, and East Asia). Moreover, we conducted univariate meta-regression to further investigate the cause of the heterogeneity.

## Results

### Search results

**Fig. 1:**
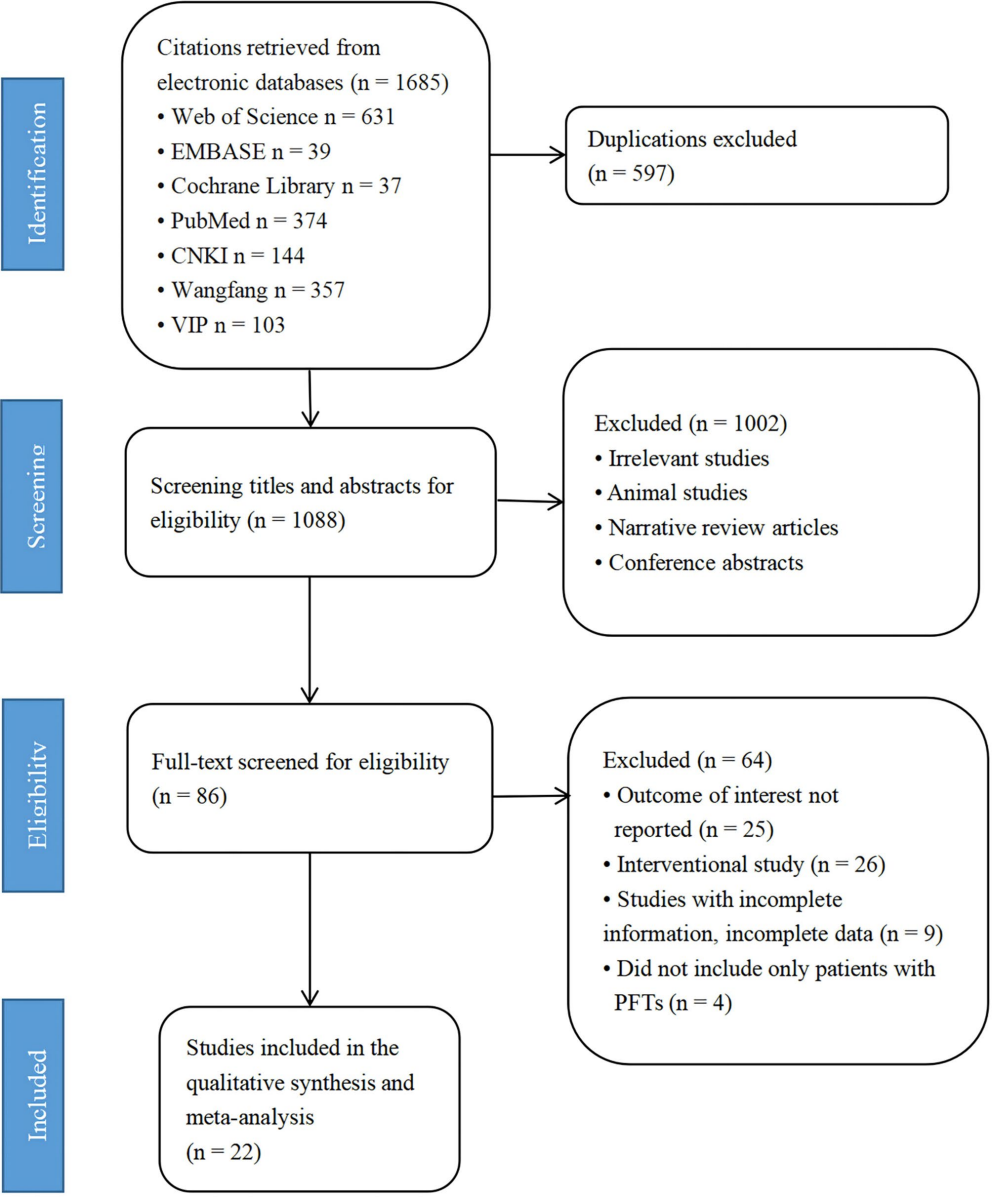
Preferred reporting items for systematic reviews and meta-analyses recommendations flowchart of search and selection of studies

The initial search identified 1,646 articles, of which 22 fulfilled the eligibility criteria and were included in the quantitative analysis. A flow chart of article selection, along with the reasons of exclusion of the articles, is presented in Fig. 1.

### Study characteristics

A total of 17 cross-sectional studies and 5 case-control studies were included in the analysis. All studies enrolled participants from clinical settings, except for the two epidemiological studies that analyzed population-level data [5, 21]. The sample size in these studies ranged from 11 to 17,281 participants. Of the 22 articles, 10 were published in Chinese and 12 in English. Seven studies [4, 7, 8, 10, 11, 22, 23] used instrumental and objective assessments, including video fluoroscopic swallowing study (VFSS) or fiberoptic endoscopic evaluation of swallowing (FEES). Among the remaining 15 studies, 8 [5, 21, 24–29] did not specify the assessment method, whereas the remaining 7 [1, 30–35] used screening tools, such as the water swallowing test (WST), repetitive saliva swallowing test (RSST), standardized swallowing assessment (SSA), clinical assessment of paediatric neurogenic dysphagia (CAPND), the dysphagia subscale of the Chinese scale of clinical neurologic deficit of stroke patients, and the dysphagia subscale of cerebellar mutism syndrome scale to detect dysphagia. Among the aforementioned screening tools, WST and SSA were the most commonly used. Table 1 summarizes the characteristics of the 22 studies.

**Table 1:** Characteristics of the included studies

First author [ref.]	Year	Study design	Country	Sample Size, No. (M/F)	Age, y^a^	Number of patients with dysphagia	Tumor site (*n*)	Tumor type (*n*)	Assessment method for dysphagia	Methodological quality
Wright [22]	2023	Cross-sectional	UK	70	4.3 (0.8–19.8)^b^	11	4th ventricle (3/23)^d^; Cerebellar hemisphere (0/19)^d^; Brain stem (2/15)^d^; CPA (6/13)^d^	Medulloblastoma (3/14)^e^; Ependymoma (4/12)^e^; Embryonal tumour with multilayer rosettes (0/2)^e^; Glioma (3/33)^e^; Atypical teratoid rhabdoid tumour, (0/1)^e^; Other (0/8)^e^	VFSS	High
Li [30]	2022	Case-control	China	228 (Anterior cranial fossa 104; Middle cranial fossa 66; Posterior fossa 58)	-	Anterior cranial fossa 3; Middle cranial fossa 7; Posterior fossa 14	Posterior fossa (58)	Meningioma (84); Metastatic tumor (55); Glioma (49); Other (40)	WST	High
Nasrollahi [24]	2022	Cross-sectional	USA	798 (409/389)	-	130	Unclear	Acoustic neuroma (798)	-	High
Li [21]	2021	Case-control	China	98 (Anterior cranial fossa 32; Middle cranial fossa 38; Posterior fossa 28)	No dysphagia: 55.14 ± 11.47 Dysphagia: 52.63 ± 17.90	Anterior cranial fossa 0; Middle cranial fossa 5; Posterior fossa 3	Posterior fossa (28)	Meningioma (42); Metastatic tumor (28); Glioma (14); Other (14)	WST	High
Zhang [32]	2021	Case-control	China	147 (Posterior fossa 39; Non posterior fossa 108)	No dysphagia: 52 (45–63)^c^ Dysphagia: 59 (50–65)^c^	Posterior fossa 18; Non posterior fossa 13	Posterior fossa (39)	Unclear	SSA	High
Alkins [5]	2021	Cross-sectional	Canada	1456 (657/799)	50.3 ± 15.3	285	Unclear	Vestibular schwannoma (1456)	-	High
Ricci [33]	2021	Cross-sectional	Italy	30 (19/11)	8 (3–10)^c^	9	Left cerebellar hemisphere (9); Right cerebellar hemisphere (6); Midline (vermis/4th ventricle, 15)	Medulloblastoma (6); Pilocytic astrocytoma (15); Ependymoma (3); Difused glioma (3); Cavernoma (1); Papilloma (1); Ganglioglioma (1)	Cerebellar mutism syndrome scale	Moderate
Yang [34]	2020	Case-control	China	91 (61/30)	-	32	Cerebellar midline (61); Cerebellar hemisphere (30)	Medulloblastoma (45); Pilocytic astrocytoma (22); Ependymoma (10); Glioma (6); Other (8)	Dysphagia subscale of Chinese scale of the clinical neurologic deficit of stroke patients	High
Goethe [4]	2020	Case-control	USA	197 (112/85)	No dysphagia: 7.2 ± 4.6 Dysphagia: 4.5 ± 3.4	43	Cerebellum (8/82)^d^; 4th ventricle (25/96)^d^; Brainstem (6/13)^d^; CPA (4/9)^d^	Juvenile pilocytic astrocytoma (7/69)^e^; Medulloblastoma (13/66)^e^; Ependymoma (12/33)^e^; Atypical teratoid/rhabdoid tumor (4/10)^e^; Other (5/19)^e^	VFSS	High
Lapa [10]	2020	Cross-sectional	Germany	26 (13/13)	49 ± 14	15	Intraaxial paramedian (2); Petroclival (3); Tentorium (4); Cerebellum (1); Petrous bone and Foramen magnum (2); CN VIII (2); Median intraaxial cystic (2); CN IX u. X (1); Cerebellumintraaxial (2); CPA (5); Clivus (1); Intrameatal (1)	Meningioma (9); Vestibular schwannoma (6); Metastases (5); other (6)	WST and FEES	High
Wang [35]	2018	Cross-sectional	China	41 (15/26)	49.6 ± 12.9	25	Cerebellum and 4th ventricle (20); CPA (20); Petroclival (1)	Unclear	RSST and WST and SSA	High
Chen [25]	2017	Cross-sectional	China	106 (59/47)	33.6 (9–57)^b^	1	4th ventricle (81); Brainstem (25)	Medulloblastoma (53); Pilocytic astrocytoma (3); Ependymoma (23); Cerebral cavernous angiomas (18); Choroid plexus papilloma (2); other (7)	-	Moderate
Lee [8]	2016	Cross-sectional	Korea	183 (108/75)	-	39	Unclear	Medulloblastoma (71); Pilocytic astrocytoma (49); Ependymoma (26); Atypical teratoid rhabdoid tumor (12); Vestibular schwannoma (6); Choroid plexus papilloma (5); Other (14)	VFSS	High
Zhang [26]	2012	Cross-sectional	China	31 (17/14)	26.5 (14–62)^b^	1	4th ventricle (31)	Medulloblastoma (6); Pilocytic astrocytoma (4); Ependymoma (12); Angioblastoma (5); Choroid plexus papilloma (2); Other (2)	-	Moderate
Wu [27]	2012	Cross-sectional	China	115 (79/36)	13.5 (1–34)^b^	11	4th ventricle (94); Cerebellum hemisphere (19); CPA (2)	Medulloblastoma (115)	-	Moderate
Ward [21]	2012	Cross-sectional	USA	17,281	50.4 (18–97)^b^	443	Unclear	Vestibular schwannoma (17,281)	-	High
Mei [7]	2011	Cross-sectional	Australia	27 (18/9)	6.7 (2.0–13.8)^b^	9	4th ventricle (26); Brainstem (22); Cerebellar midline (12); Cerebellum left hemisphere (8); Cerebellum right hemisphere (3)	Medulloblastoma (11); Pilocytic astrocytoma (9); Ependymoma (4); Ganglioglioma (1); Germinoma (1); Atypical teratoid/rhabdoid tumour (1)	VFSS	High
Starmer [23]	2011	Cross-sectional	USA	181 (76/105)	49 (20–81)^b^	57	CPA (181)	Acoustic neuromas (168); Other (Meningioma, Hamartoma, Rhabdomyoma, and Epidermoid, 13)	VFSS	High
Chen [29]	2010	Cross-sectional	China	44 (24/20)	28.3 (2–56)^b^	3	4th ventricle (44)	Medulloblastoma (9); Pilocytic astrocytoma (2); Ependymoma (25); Angioblastoma (3); Choroid plexus papilloma (2); Other (3)	-	Moderate
Deng [28]	2009	Cross-sectional	China	84 (48/36)	5.4 (3–7)^b^	6	4th ventricle (84)	Glioma (84)	-	High
Morgan [1]	2008	Cross-sectional	UK	11 (9/2)	8.0 ± 2.9	8	4th ventricle, Brainstem (1); 4th ventricle, Cerebellum (6); Cerebellum (4)	Pilocytic astrocytoma (4); Medulloblastoma (6); Choroids plexus papillpma (1)	Clinical assessment of paediatric neurogenic dysphagia	High
Newman [11]	2006	Cross-sectional	USA	24 (15/9)	5.9 ± 5.3	7	CPA (7); 4th ventricle (4); Brain stem (5); Midbrain (1); Cerebellum (1); Unclear (6)	Ependymoma (12); Malignant fibrous histiocytoma (1); Glioma (2); Astrocytoma (3); Medulloblastoma (2); Unclear (4)	VFSS	High

M = male; F = female; UK = United Kingdom; USA = United States; CPA = cerebellopontine angle; CN = cranial nerve; VFSS = video fluoroscopic swallowing study; WST = water swallowing test; SSA = standard swallowing assessment; FEES = fiberoptic endoscopic evaluation of swallowing; RSST = Repeat saliva swallowing test.

^a^Values are expressed as mean ± SD, unless indicated otherwise.

^b^Median (range).

^c^Median (interquartile range).

^d^(Number of patients with dysphagia after surgery at a specific tumor site/Number of patients at a specific tumor site).

^e^(Number of patients with dysphagia after surgery for a specific tumor type/Number of patients with a specific tumor type).

### Risk of bias

**Fig. 2:**
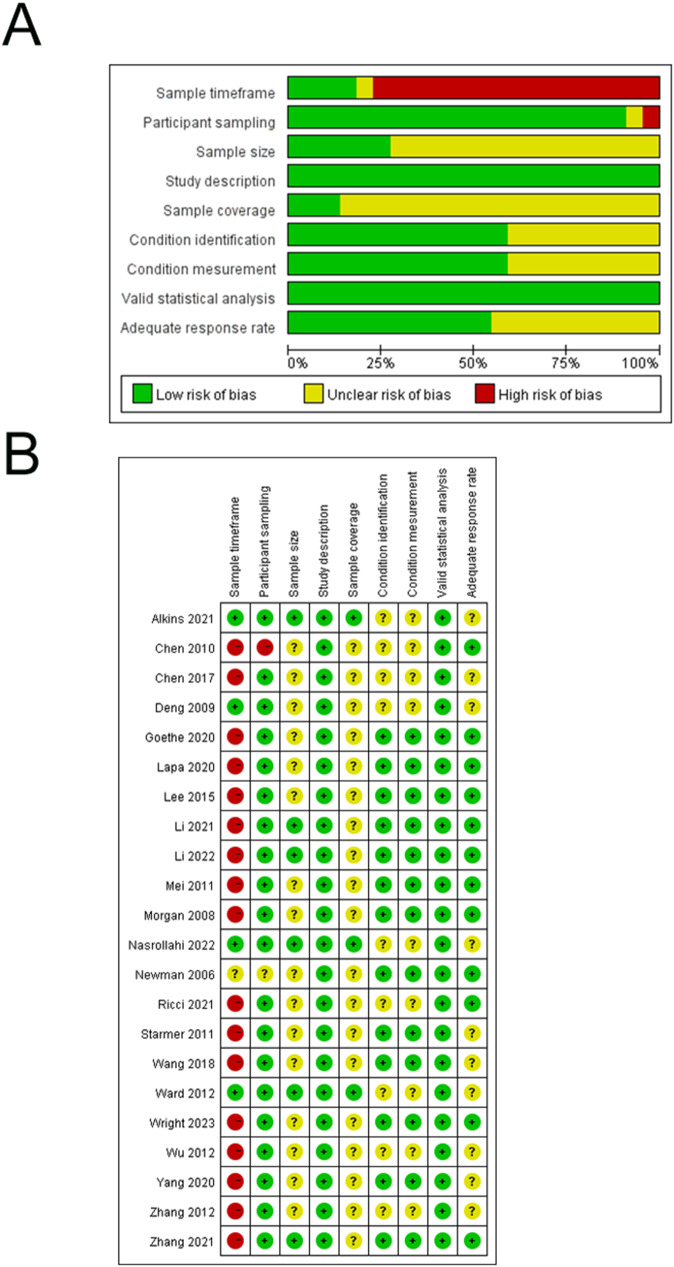
Risk of bias. **A**: Bar chart demonstrating the authors’ judgements on each methodological quality item presented as percentages for all included studies. **B**: Summary of authors’ judgements on each risk of bias item for all included studies (*n* = 22)

The mean total quality score was 13.41 ± 1.53, with 17 (77.3%) studies exhibiting a high quality and the remaining 5 (22.7%) demonstrating a moderate risk of bias. Furthermore, 17 (77.3%) of the 22 studies recruited participants from a single healthcare center, resulting in a high risk of bias related to the sample timeframe. Most studies had unclear risk of bias on the sample size or sample coverage. Figure 2 provides a summary of the risk of bias assessment for the included studies.

### Prevalence of dysphagia following PFT resection

**Fig. 3:**
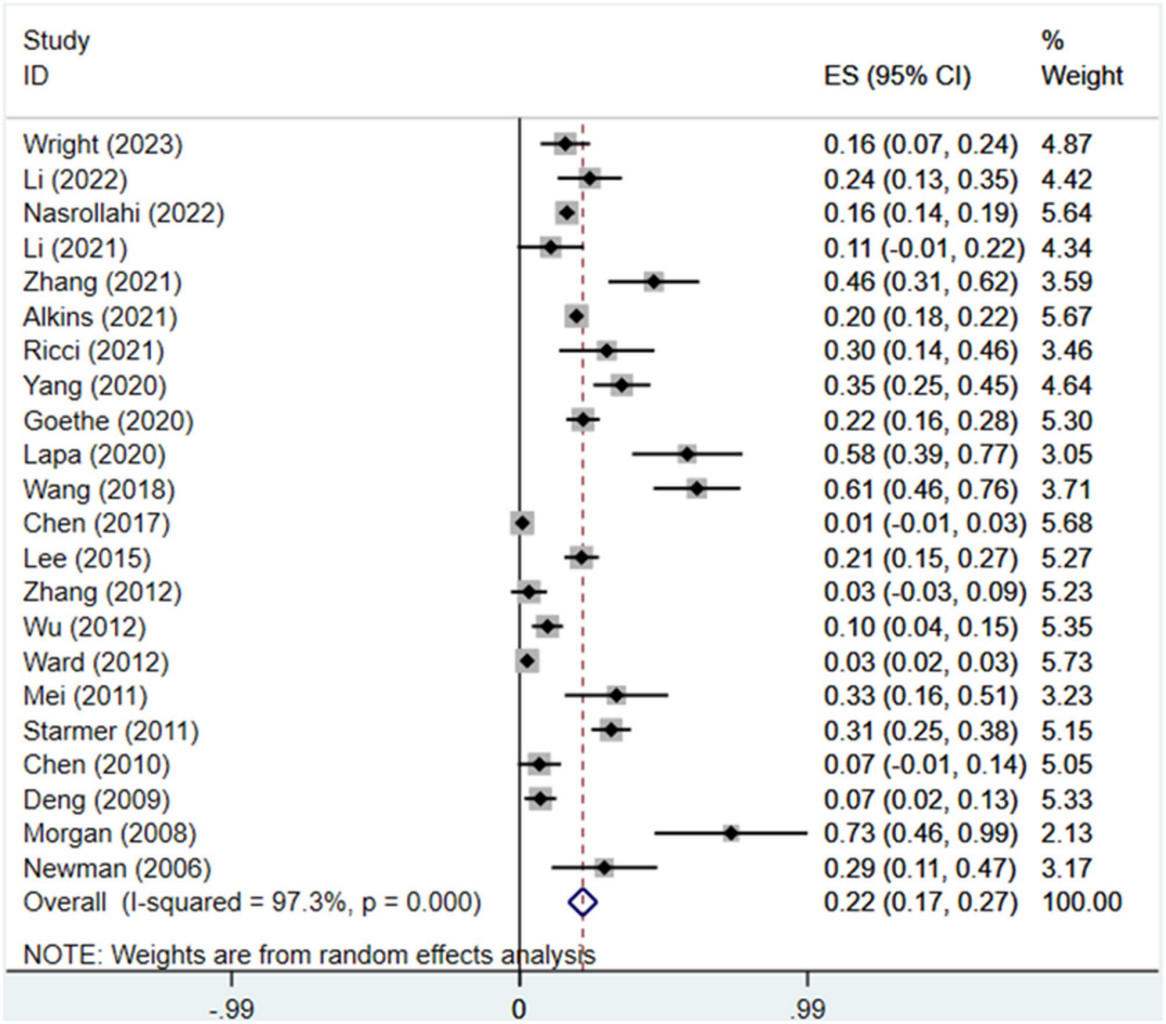
The forest plot of the overall estimation of the prevalence of dysphagial following posterior fossa tumor resection based on the random efects model. ES, effect size; CI, confidence interval

In total, the 22 studies included 20,921 participants who had undergone PFT resection. The prevalence rate of postoperative dysphagia showed a wide range (0.9–72.7%) among the included studies. The random-effects meta-analysis resulted in a summary estimate of 21.7% (95% CI: 16.9–26.6) (Fig. 3). There was significant heterogeneity among the included studies (*I*^2^ = 97.3%; *P* < 0.001).

### Subgroup and meta-regression analyses

The subgroup analyses were conducted according to the study design, publication year, age, assessment method of dysphagia, and the geographical region of the study population. Table 2 presents the summary of subgroup analyses and heterogeneity assessments. The subgroup analyses demonstrated high heterogeneity among the included studies. Accordingly, meta-regression analyses were performed to explore the sources of the heterogeneity. As shown in Table 2, the meta-regression analyses showed that the age of the participants (*P* < 0.001), assessment methods for dysphagia (*P* = 0.004), and geographical region of the study population (*P* = 0.001) were significantly associated with heterogeneity among the included studies.

**Table 2:** Subgroup and meta-regression analyses of the prevalence of dysphagia following PFT resection

Subgroup	Heterogeneity test		Effects model	Pooled prevalence rate % (95% CI)	Coefficient (95% CI)	SE	*P* > | t |
	I^2^	*P*					
Study design					0.177 (-0.086–0.440)	0.126	0.175
Cross-sectional (*n* = 17)	97.5%	< 0.001	Random effects	20.0 (14.7–25.2)			
Case–control (*n* = 5)	78.1%	0.001	Random effects	26.7 (17.1–36.3)			
Year of publication					0.114 (-0.170–0.398)	0.136	0.412
2006–2013 (*n* = 9)	93.7%	< 0.001	Random effects	16.8 (9.0–24.5)			
2014–2023 (*n* = 13)	96.4%	< 0.001	Random effects	25.7 (18.6–32.7)			
Sample size					0.106 (-0.103–0.314)	0.100	0.302
10–99 (*n* = 14)	90.9%	< 0.001	Random effects	28.8 (19.3–38.4)	0.183 (-0.051–0.416)	0.111	0.118
100–999 (*n* = 5)	97.8%	< 0.001	Random effects	18.1 (7.2–29.0)	0.077 (-0.188–0.341)	0.126	0.551
> 1000 (*n* = 3)	99.3%	< 0.001	Random effects	10.6 (-2.4–23.5)	Reference		
Age, y					0.272 (0.151–0.392)	0.058	< 0.001
< 18 (*n* = 9)	84.5%	< 0.001	Random effects	25.9 (17.8–33.9)	Reference		
≥ 18 (*n* = 9)	98.6%	< 0.001	Random effects	27.6 (18.4–36.8)	0.009 (-0.159–0.178)	0.080	0.908
Uncategorized (*n* = 4)	71.7%	0.014	Random effects	4.7 (0.1–9.2)	-0.221 (-0.428 – -0.013)	0.099	0.038
Assessment method					0.329 (0.117–0.541)	0.101	0.004
Simple symptom screening (*n* = 2)	0.0%	0.596	Random effects	33.8 (25.4–42.2)	Reference		
Bedside assessments (*n* = 5)	90.5%	< 0.001	Random effects	41.4 (20.3–62.4)	0.060 (-0.194–0.314)	0.138	0.628
Instrumental and objective assessments (*n* = 7)	74.0%	0.001	Random effects	27.2 (20.2–34.1)	-0.046 (-0.285–0.193)	0.114	0.689
Undeclared (*n* = 8)	98.2%	< 0.001	Random effects	8.4 (2.6–14.1)	-0.246 (-0.477– -0.015)	0.110	0.038
Region					0.408 (0.202–0.614)	0.098	0.001
North America (*n* = 6)	99.0%	< 0.001	Random effects	19.4 (9.4–29.4)	-0.212 (-0.447–0.023)	0.112	0.074
East Asia (*n* = 11)	94.4%	< 0.001	Random effects	19.0 (11.0–26.9)	-0.211 (-0.467–0.045)	0.122	0.100
Europe (*n* = 4)	89.5%	< 0.001	Random effects	42.2 (17.3–67.0)	Reference		
Australia (*n* = 1)	-	-	-	33.3 (15.6–51.1)	-0.075 (-0.535–0.386)	0.219	0.738

CI = confidence interval; SE = standard error.

**Fig. 4:**
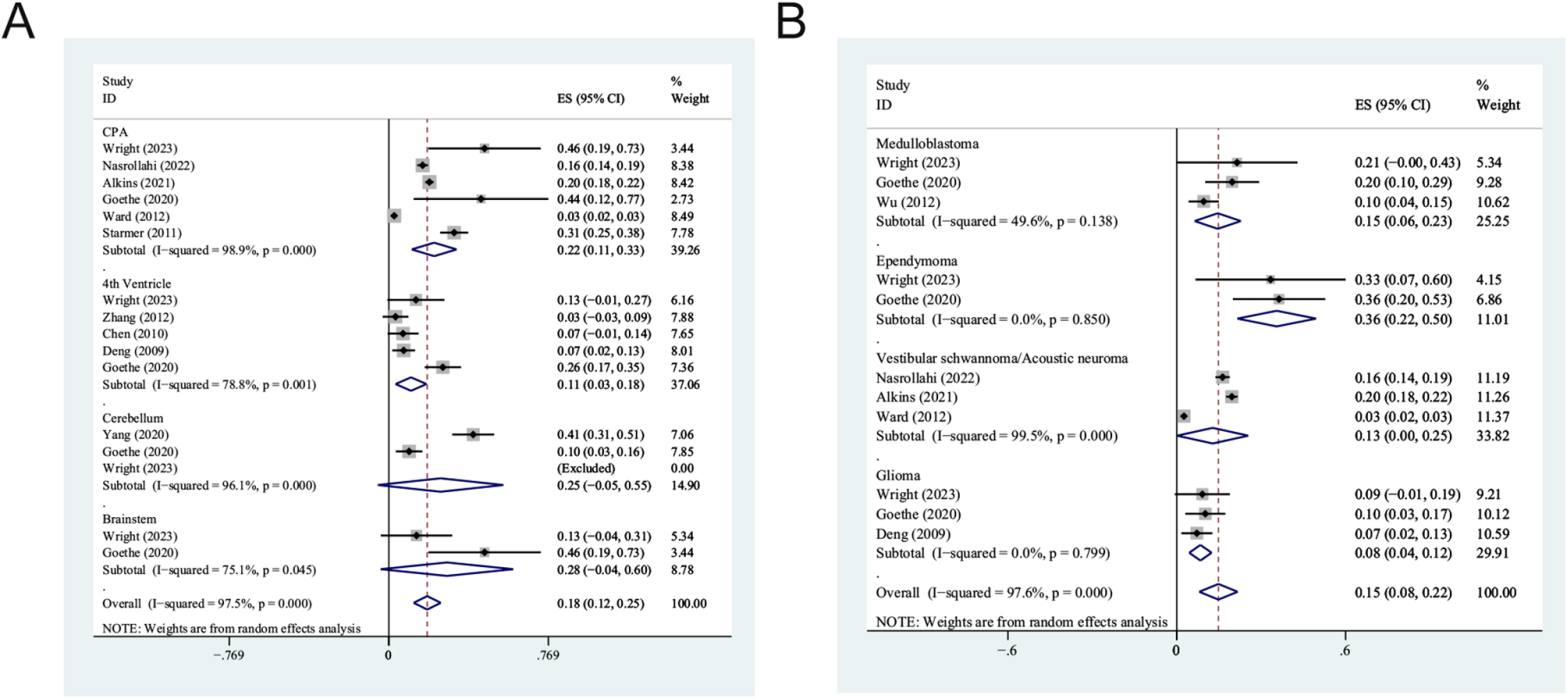
The forest plot of the overall estimation of the prevalence of dysphagia following PFT resection based on different tumor sites (**A**) and types (**B**). CPA = cerebellopontine angle; ES, effect size; CI, confidence interval

Given that two studies showed the incidence of postoperative dysphagia for each tumor site or tumor type, while other studies either only focused on a tumor site (e.g., CPA, fourth ventricle) or a tumor type (e.g., medulloblastoma, acoustic neuroma), or did not clarify the specific site and type of PFTs, it was difficult to conduct subgroup analysis based on tumor site or tumor type among all included studies. Therefore, we excluded studies that did not clarify tumor sites or types, reorganized the data, and then conducted subgroup analysis according to tumor sites and tumor types (Table 3). Figure 4 provides the forest plots of the pooled prevalences of dysphagial following PFT resection based on different tumor sites and types.

**Table 3:** Subgroup analyses of the prevalence of dysphagia following PFT resection according to tumor site and tumor type

Subgroup	Heterogeneity test		Effects model	Pooled prevalence rate % (95% CI)
	I^2^	*P*		
Tumor site				
CPA (*n* = 6 )	98.9%	< 0.001	Random effects	21.8 (11.0–32.7)
4th ventricle (*n* = 5 )	78.8%	0.001	Random effects	10.7 (3.2–18.1)
Cerebellum (*n* = 3)	96.1%	< 0.001	Random effects	25.0 (–5.3–55.2)
Brainstem (*n* = 2)	75.1%	0.045	Random effects	28.0 (–4.0–60.0)
Tumor type				
Medulloblastoma (*n* = 3)	49.6%	0.138	Random effects	14.6 (6.4–22.8)
Ependymoma (*n* = 2)	0.0%	0.850	Random effects	35.5 (21.6–49.5)
Vestibular schwannoma/Acoustic neuroma (*n* = 3)	99.5%	< 0.001	Random effects	12.8 (0.1–25.4)
Glioma (*n* = 3)	0.0%	0.799	Random effects	8.4 (4.4–12.4)

CPA = cerebellopontine angle; CI = confidence interval; SE = standard error.

## Discussion

This review quantitatively synthesized the current evidence on the prevalence of dysphagia following PFT resection. The analysis included 22 studies with 20,921 participants, and found a pooled global prevalence of 21.7%, which is slightly higher than the estimated prevalence rate of approximately 20% in the general population reported in a national health interview survey [36]. Rajat et al. [37]. reported the global prevalence of oropharyngeal dysphagia in different populations to be 43.8%. Our findings are not consistent with those of previous studies, which can be attributed to the inclusion of a large number of stroke and dementia patients in the systematic review and meta-analysis conducted by Rajat et al., while the present study focused on patients who had undergone PFT resection.

Due to the deep anatomical position and small space of the posterior cranial fossa, the posterior cranial nerves (IX–XII) located adjacent to the PFT are vulnerable to injury and traction during the prolonged duration of tumor resection, resulting in hypoesthesia, loss of function of the glossopharyngeal nerve, and atrophy of the lingual muscles [35]. Moreover, postoperative brain edema may compress the aforementioned nerves and lead to dysphagia [38]. In addition, the use of tracheal intubation during the perioperative period can also affect the occurrence of dysphagia. Studies have shown that post-extubation dysphagia is common in patients admitted to the intensive care unit [17]. Our study found that patients who have undergone PFT removal are at a high risk of postoperative dysphagia. Consequently, clinicians should pay attention to dysphagia in these patients, as aspiration pneumonia caused by dysphagia is one of the leading causes of death [9]. The other complications of dysphagia include bronchospasm, chronic malnutrition and weight loss, muscle wasting, and dehydration [9]. Dysphagia is a primary reason for delayed hospital discharge and increased need for healthcare services [18, 19, 39–41]. Therefore, it is essential to identify dysphagia early in patients who have undergone PFT removal.

Dysphagia, a symptom of swallowing impairment, can occur in cases of swallowing dysfunction [9]. The lack of uniform definitions of dysphagia and significant methodological variability among the included studies made it difficult to perform synthesis of the prevalence data. In the present review, eight different definitions of dysphagia were used by the included studies. Based on the assessment methods for dysphagia, we divided the studies into four subgroups: simple symptom screening, bedside assessments (WST, RSST, SSA, and CAPND), instrumental and objective assessments (VFSS and FEES), and undeclared methods. The prevalence of dysphagia was 33.8% (95% CI: 25.4–42.2), 41.4% (95% CI: 20.3–62.4), 27.2% (95% CI: 20.2–34.1), and 8.4% (95% CI: 2.6–14.1) in these subgroups, respectively. In the simple symptom screening studies, patients were evaluated for dysphagia based on the presence or absence of symptoms related to difficulty in eating or drinking water, which is a convenient and suitable method for the rapid identification of the risk of dysphagia in clinical practice. The bedside clinical swallowing examination is conducted by trained healthcare professionals, typically speech-language pathologists [9]. This test assesses the swallowing with foods and liquids of different consistencies, and provides additional information about the patient’s cognition, phonation, cranial nerve function involved in swallowing, speech intelligibility, and cough strength to evaluate the patient’s ability to handle secretions [9, 42]. However, bedside assessment is inappropriate to monitor for silent aspiration or motility issues; for these reasons, instrumental and objective assessments may be performed. VFSS and FEES enable the visualization of the swallowing mechanism and provide detailed information on the swallowing function [9]. They involve the use of specialized equipment, which require the presence of an imaging specialist and a speech-language pathologist. However, these assessment methods are invasive and may damage the body. Therefore, VFSS and FEES should not be used as initial diagnostic tools if a structural cause of dysphagia is suspected and there is no concern for aspiration. In the present study, the meta-regression analyses showed that the difference in the assessment methods for dysphagia was one possible cause of heterogeneity among the studies (*P* = 0.004). The results indicate the importance of selecting an appropriate assessment method for dysphagia following PFT resection, suggesting that clinicians should conduct instrumental and objective assessments of swallowing in a timely manner, where appropriate, in addition to the bedside assessment [9].

The prevalence of dysphagia varies with age. Rajati et al. reported that the prevalence of oropharyngeal dysphagia increased with increasing age [37]. Wilkins et al. found that patients with dysphagia in primary care were more likely to be older than those without dysphagia (mean ages of 48.1 and 45.7 years in patients with and without dysphagia, respectively; *P* = 0.001) [43]. Other studies have identified a prevalence of dysphagia of 40–50% among older individuals residing in long-term care facilities [44]. A previous survey [36] showed that a significant proportion of respondents identified “advancing age” as an etiology for their swallowing problems. This may be because of the deteriorating swallowing reflex and body function of older individuals, which leads to the loss of muscle strength and nerve functions that control eating, thereby predisposing to dysphagia [30]. Although the aforementioned studies have focused on the evaluation and management of dysphagia in adults, the prevalence of dysphagia in the pediatric population also requires attention, as dysphagia is a common pediatric disease, although it is not as common in children as in the older population [9]. This review found that the estimated pooled prevalence of dysphagia was 25.9% (95% CI: 17.8–33.9) in pediatric patients and 27.6% (95% CI: 18.4–36.8) in adults. These results suggest that age was a source of heterogeneity among the included studies (*P* < 0.001). The current data from adult patients were not restricted to older patients, which may explain the lack of significant difference in the prevalence of dysphagia between children and adults. Dysphagia in the pediatric population also requires early detection and treatment to prevent malnutrition and delayed developmental milestones, which may negatively impact the patient health.

Considering the differences between adults and children with the respect to the tumor types and surgical strategies of PFTs, we conducted subgroup analyses of tumor site and tumor type separately. Among all the structures involved in the included studies, the highest prevalence of dysphagia after PFTs resection was in the brainstem (28.0%, 95% CI: − 4.0–60.0), followed by the cerebellum (25.0%, 95% CI: − 5.3–55.2), CPA (21.8%, 95% CI: 11.0–32.7), and the fourth ventricle (10.7%, 95% CI: 3.2–18.1). Tumors in the brainstem may compromise the lower cranial nerves, affecting pharyngeal function and swallowing ability [7]. Goethe and colleague reported that patients who developed dysphagia post-operatively were more likely to have brainstem involvement [4]. PFTs resection in brainstem is more challenging, leading to a risk for injury to key structures and has been associated with postoperative dysphagia. In addition to site, tumor type is also one of the factors contributing to postoperative dysphagia. The results of subgroup analysis showed that the pooled prevalence of postoperative dysphagia in ependymoma, medulloblastoma, vestibular schwannoma/acoustic neuroma, and glioma subgroup was 35.5% (95% CI: 21.6–49.5), 14.6% (95% CI: 6.4–22.8), 12.8% (95% CI: 0.1–25.4), and 8.4% (95% CI: 4.4–12.4), respectively. Previous report has concluded that ependymoma were associated with increased risk of postoperative dysphagia [8]. A possible reason is that ependymomas and medulloblastomas are closely related to cranial nerves and the dorsal brainstem [22]. The lower cranial nerves involved in swallowing have a greater risk of injury in surgical removal of ependymomas and medulloblastomas compared to other PFTs.

It should be noted that our study only summarized the prevalence rate of postoperative dysphagia in different tumor sites and types, and provided insufficient evidence regarding the risk factors for dysphagia in patients undergoing surgery for PFTs. Moreover, there are many other factors that were not included in this study. For example, radiation intervention for head and neck cancer has been recognized as a potentially dose-limiting toxicity to swallowing function [45]. Neuropathy and fibrosis of the oral and pharyngeal musculature may persist for a long time after the completion of treatment, ultimately impairing the motor function of key swallowing structures and leading to long-term dysphagia [45]. Previous study has pointed that half of head and neck cancer patients undergoing radiationtherapy may have significant dysphagia [46]. Given the importance associated with management of swallowing dysfunction, further research is needed to explore the risk factors for dysphagia after PFTs resection.

The meta-regression analysis showed that the geographical region of the study population (*P* = 0.001) was a significant source of heterogeneity among the included studies. According to the subgroup analyses based on different regions, the highest prevalence of dysphagia was found in Europe (42.2%, 95% CI: 17.3–67.0), followed by Australia (33.3%, 95% CI: 15.6–51.1), North America (19.4%, 95% CI: 9.4–29.4), and East Asia (19.0%, 95% CI: 11.0–26.9). Rajati et al. also confirmed substantial variations in the pooled prevalence of oropharyngeal dysphagia between different regions and countries [37]. One explanation for this variation is the population structure in different countries of the world [37]. In addition, the differences in the use of advanced techniques may be one of the reasons for the variations in the results across different regions. In this study, VFSS and FEES were used more frequently in North American and European studies. However, nearly half of the Asian studies did not specify the assessment methods for dysphagia. It is necessary to carefully evaluate the prevalence of dysphagia in different regions to emphasize that clinicians should pay attention to this symptom and its consequences.

Despite the rigorous methodology of this review, our study had certain limitations. First, most of the included studies had a relatively short follow-up and a retrospective or cross-sectional design, leading to the possibility of high levels of selection bias. Second, there was significant heterogeneity among the included studies. Furthermore, some of the included studies had small sample sizes and variations in the assessment protocols. In addition, combining pediatric and adult patients may complicate the analysis due to significant differences the nature of PFTs. A focus on pediatric or adult patients alone might provide clearer insights. Despite these limitations, this review represents the first known meta-analysis of the prevalence of dysphagia following PFT resection. Our results have significant implications for clinical practice as our findings emphasize that clinicians should pay attention to the occurrence of dysphagia in patients who have undergone PFT and provide foundations for targeted treatment strategies.

## Conclusions

This systematic review and meta-analysis revealed a high prevalence of dysphagia following PFT resection. Individuals who have undergone PFT resection are at a high risk of dysphagia and should be identified early through screening methods. Multidisciplinary diagnosis and treatment of dysphagia is required to improve the outcomes in the early stages after PFT resection.

### Electronic supplementary material

Below is the link to the electronic supplementary material.

Supplementary Material 1

## Data Availability

The datasets generated and/or analyzed during the current study are available from the corresponding author on reasonable request.
