# Supplementary material for: Prevalence of dysphagia following posterior fossa tumor resection: a systematic review and meta‑analysis
Source: BMC Cancer. 2024 Jul 25;24:896. doi: 10.1186/s12885-024-12656-1 (PMC11282789; doi:10.1186/s12885-024-12656-1)

# **Supplementary Material**

# **S1: Search Strategies (searches were conducted on March 31, 2023)**

PubMed search strategy

((((((((((((((posterior fossa tumor) OR (brain tumor)) OR (medulloblastoma[MeSH Terms])) OR (astrocytoma[MeSH Terms])) OR (ependymoma[MeSH Terms])) OR (glioma[MeSH Terms])) OR (cerebellopontine angle tumor[Title/Abstract])) OR (CPA[Title/Abstract])) OR (brain stem neoplasm[Title/Abstract])) OR (brain stem tumor[Title/Abstract])) OR (the fourth ventricular tumor[Title/Abstract])) OR (tumor of the fourth ventricle[Title/Abstract])) OR (lower cranial nerve deficits)) OR (acoustic neurinoma[MeSH Terms])) AND ((((((((dysphagia[MeSH Terms]) OR (difficulty swallowing[Title/Abstract])) OR (dysphagy[Title/Abstract])) OR (impaired swallowing[Title/Abstract])) OR (swallowing dysfunction[Title/Abstract])) OR (swallowing impairment[Title/Abstract])) OR (deglutition disorder[Title/Abstract])) OR (deglutition difficulty[Title/Abstract]))

Cochrane library search strategy

(posterior fossa tumor OR acoustic neurinoma OR brain tumor OR glioma OR (medulloblastoma OR astrocytoma OR ependymoma OR posterior fossa brain tumor OR cerebellopontine angle tumor OR CPA tumor OR brain stem neoplasm OR brain stem tumor OR the fourth ventricular tumor OR tumor of the fourth ventricle OR lower cranial nerve deficits) in Title Abstract Keyword AND (dysphagia OR difficulty swallowing OR dysphagy OR impaired swallowing OR swallowing dysfunction OR swallowing impairment OR deglutition disorder OR deglutition difficulty) in Title Abstract Keyword

Web of science search strategy

(posterior fossa tumor OR acoustic neurinoma OR brain tumor OR glioma OR (medulloblastoma OR astrocytoma OR ependymoma OR posterior fossa brain tumor OR cerebellopontine angle tumor OR CPA tumor OR brain stem neoplasm OR brain stem tumor OR the fourth ventricular tumor OR tumor of the fourth ventricle OR lower cranial nerve deficits) in Topic AND (dysphagia OR difficulty swallowing OR dysphagy OR impaired swallowing OR swallowing dysfunction OR swallowing impairment OR deglutition disorder OR deglutition difficulty) in Topic

Embase search strategy


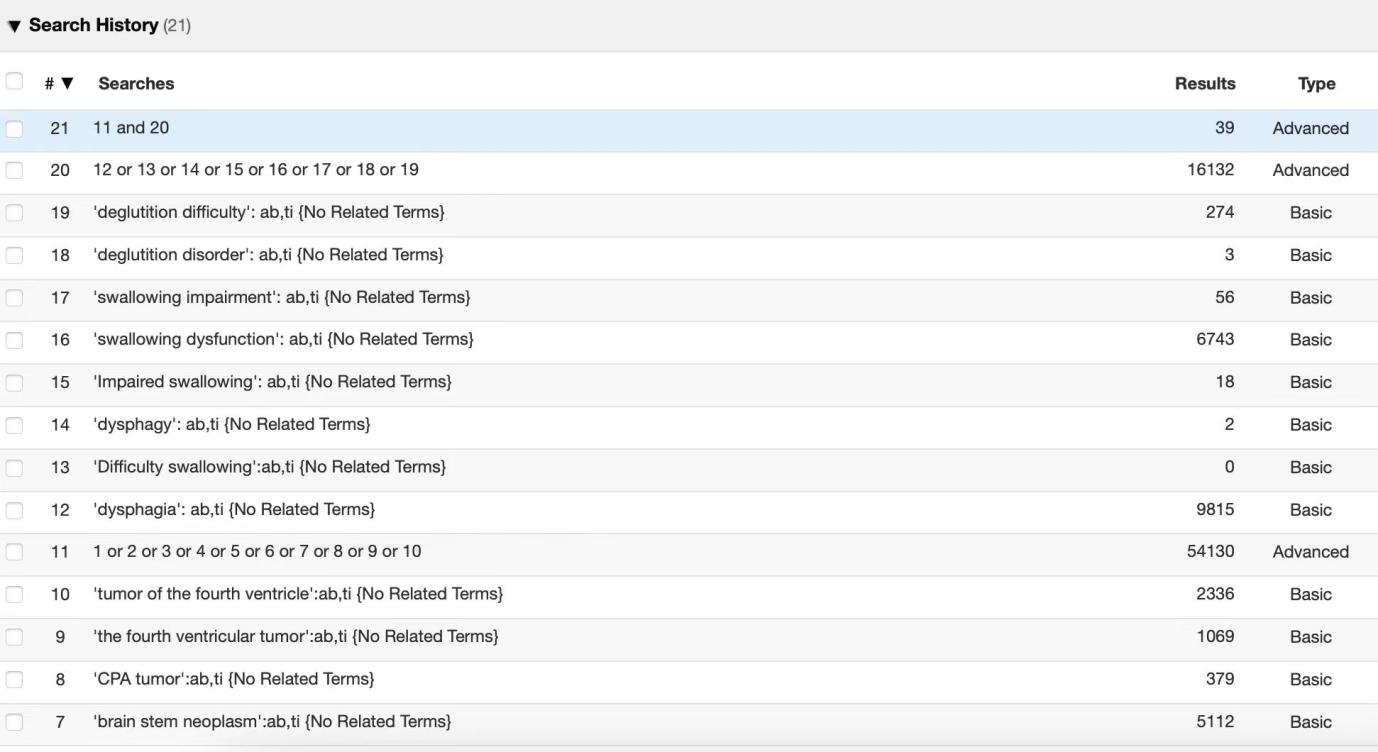


CNKI database

SU%= (posterior fossa tumor OR acoustic neurinoma OR brain tumor OR glioma OR (medulloblastoma OR astrocytoma OR ependymoma OR posterior fossa brain tumor OR cerebellopontine angle tumor OR CPA tumor OR brain stem neoplasm OR brain stem tumor OR the fourth ventricular tumor OR tumor of the fourth ventricle OR lower cranial nerve deficits) AND SU%= (dysphagia OR difficulty swallowing OR dysphagy OR impaired swallowing OR swallowing dysfunction OR swallowing impairment OR deglutition disorder OR deglutition difficulty)

Wangfang database

SU%= (posterior fossa tumor OR acoustic neurinoma OR brain tumor OR glioma OR (medulloblastoma OR astrocytoma OR ependymoma OR posterior fossa brain tumor OR cerebellopontine angle tumor OR CPA tumor OR brain stem neoplasm OR brain stem tumor OR the fourth ventricular tumor OR tumor of the fourth ventricle OR lower cranial nerve deficits) AND SU%= (dysphagia OR difficulty swallowing OR dysphagy OR impaired swallowing OR swallowing dysfunction OR swallowing impairment OR deglutition disorder OR deglutition difficulty)

VIP database

### SU%= (posterior fossa tumor OR acoustic neurinoma OR brain tumor OR glioma OR (medulloblastoma OR astrocytoma OR ependymoma OR posterior fossa brain tumor OR cerebellopontine angle tumor OR CPA tumor OR brain stem neoplasm OR brain stem tumor OR the fourth ventricular tumor OR tumor of the fourth ventricle OR lower cranial nerve deficits) AND SU%= (dysphagia OR difficulty swallowing OR dysphagy OR impaired swallowing OR swallowing dysfunction OR swallowing impairment OR deglutition disorder OR deglutition difficulty)

### S2: Critical Appraisal Checklist. For each of the nine domains a score was assigned from 0-2, with 0 representing that the criteria had not been met, 1 being unclear or insufficient information, and 2 for criteria met. The domain scores were totaled (maximum obtainable 18) and the percentage of the maximum score computed for each study.


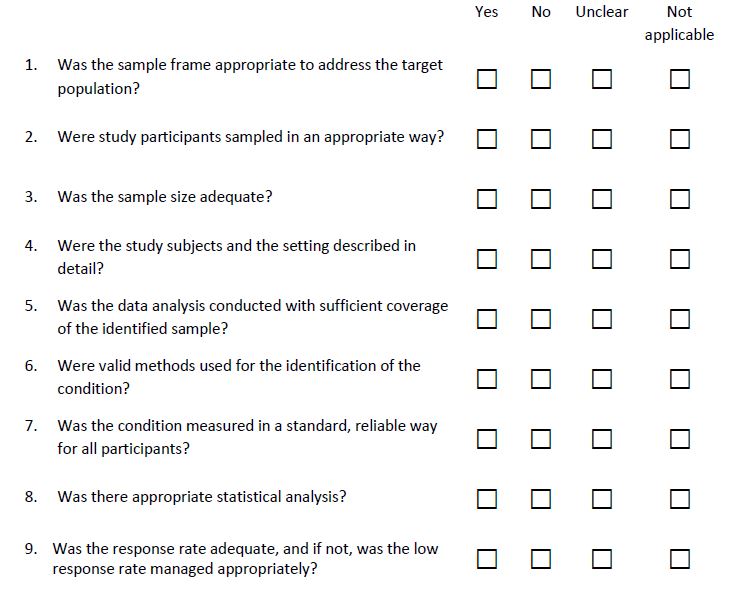

Supplement: Supplementary file 1 — Supplementary Material 1 [file 12885_2024_12656_MOESM1_ESM.docx]
